# Dual-site mixed layer-structured FA_*x*_Cs_3−*x*_Sb_2_I_6_Cl_3_ Pb-free metal halide perovskite solar cells†

**DOI:** 10.1039/d0ra00787k

**Published:** 2020-05-06

**Authors:** Yong Kyu Choi, Jin Hyuck Heo, Ki-Ha Hong, Sang Hyuk Im

**Affiliations:** Department of Chemical and Biological Engineering, Korea University 145 Anam-ro, Seongbuk-gu Seoul 02841 Korea imromy@korea.ac.kr; Department of Materials Science and Engineering, Hanbat National University 125 Dongseo-daero, Yuseong-Gu Daejeon 34158 Korea kiha.hong@hanbat.ac.kr

## Abstract

Structure engineering of trivalent metal halide perovskites (MHPs) such as A_3_Sb_2_X_9_ (A = a monovalent cation such as methyl ammonium (MA), cesium (Cs), and formamidinium (FA) and X = a halogen such as I, Br, and Cl) is of great interest because a two dimensional (2D) layer structure with direct bandgap has narrower bandgap energy than a zero dimensional (0D) dimer structure with indirect bandgap. Here, we demonstrated 2D layer structured FACs_2_Sb_2_I_6_Cl_3_ MHP by dual-site (A and X site) mixing. Thanks to the lattice-symmetry change by I–Cl mixed halide, the shortest ionic radius of Cs, and the lower solution energy due to dual-site mixing, the FACs_2_Sb_2_I_6_Cl_3_ MHP had 2D layer structure and thereby the MHP solar cells exhibited improved short-circuit current density.

## Abstract 2

Dual site mixing of FA*_x_*Cs_3−*x*_Sb_2_I_6_Cl_3_ forms stable 2D layer structure.

## Graphical abstract

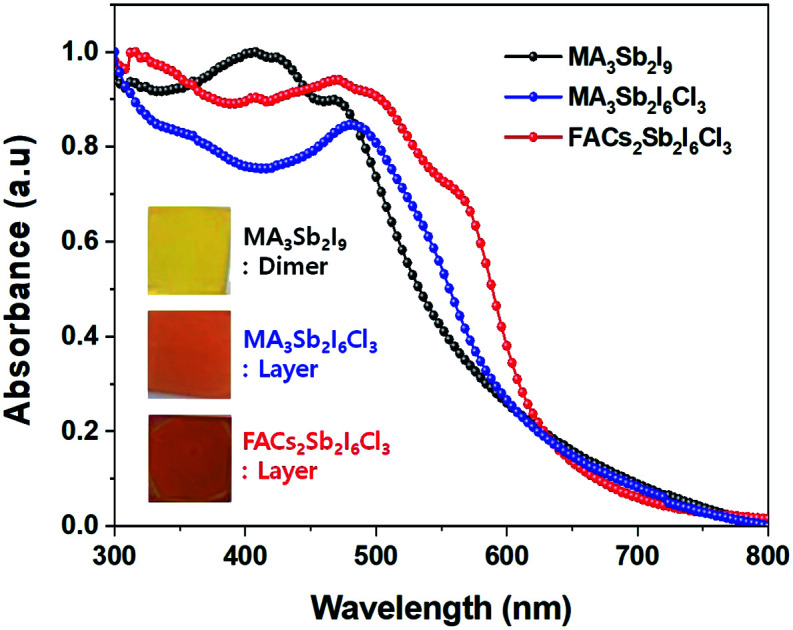

Recently, metal halide perovskite solar cells (MHP SCs) have been paid great attention because of their unique properties such as high absorptivity due to direct bandgap, long charge carrier diffusion length, small exciton binding energy, convenient bandgap tunability, and solution processability. Since Kojima *et al.* reported a liquid junction CH_3_NH_3_PbX_3_ (MAPbX_3_, X = I or Br) MHP-sensitized solar cell,^1^ intensive studies have been carried out to develop highly efficient MHP SCs.^2–8^ Accordingly, the record efficiency of a Pb-based MHP SC reached over 25% under 1 sun conditions (AM1.5G 100 mW cm^−2^).^9^ Although the MHP SCs have great potential in applications such as flexible solar cells, building integrated photovoltaics, vehicle integrated photovoltaics, and portable power generators, the Pb in the MHP SCs causes big problems for finding applications for human-friendly power generators.^10^

Hence, it is big challenging to develop efficient and stable Pb-free MHP SCs. The researches of Pb-free MHP SCs can be roughly classified to divalent metal (Sn and Ge)-based and trivalent metal (Sb and Bi)-based MHP SCs.^11^ For instance, Hao *et al.* reported on a 5.7% MASnBr_*x*_I_1−*x*_ MHP SC composed of F-doped tin oxide (FTO)/blocking TiO_2_ (bl-TiO_2_)/mesoporous TiO_2_ (*m*-TiO_2_)/MHP/2,2′,7,7′-tetrakis [*N*,*N*-di(4-methoxyphenyl) amino]-9,9′-spirobifluorene (spiro-OMeTAD)/Au.^12^ Recently Jokar *et al.* obtained 9.6% FASnI_3_ MHP SCs with improved stability by introduction of guanidinium iodide and ethylenediammonium diiodide.^13^ Heo *et al.* reported that the phase stability and durability of all inorganic CsSnI_3_ MHP SCs can be improved by addition of SnBr_2_.^14^ Krishnamoorthy *et al.* reported AGeI_3_ (A = Cs, MA, and FA ((NH_2_)_2_CH)) MHP SCs.^15^ However, the divalent metal (Sn and Ge)-based Pb-free perovskite materials have suffered from their quick oxidation to Sn^4+^/Ge^4+^ in air atmosphere.^16^

In contrast, the trivalent metal (Bi and Sb)-based MHP SCs with A_3_M_2_X_9_ (A = Cs, MA, and FA, M = Sb and Bi, X = Cl, Br, and I) crystal structure exhibit good air and thermal stability.^17–19^ However, the A_3_M_2_X_9_ MHP SCs have zero dimensional (0D) dimer structure and two dimensional (2D) layer structure. For example, Harikesh *et al.* reported that the formation energies of dimer and layer structure are −12.80 eV and −12.70 eV for Cs_3_Sb_2_I_9_ and −12.40 eV and −12.65 eV for Rb_3_Sb_2_I_9_ so that the Cs_3_Sb_2_I_9_ and Rb_3_Sb_2_I_9_ make dimer structure and layer structure, respectively.^20^ Similarly, Correa-Baena *et al.* reported that Cs_3_Sb_2_I_9_, Rb_2_Sb_2_I_9_, and K_3_Sb_2_I_9_ have dimer structure with indirect bandgap, layer structure with direct bandgap, and layer structure with indirect bandgap so their power conversion efficiencies (PCEs) are 0.03, 0.76, and 0.07%, respectively.^21^ Jiang *et al.* reported that the dimer structured MA_3_Sb_2_I_9_ is changed to the layer structured MA_3_Sb_2_I_9−*x*_Cl_*x*_ by partial substitution of I to Cl.^22^ Umar *et al.* also reported that the dimer structured MA_3_Sb_2_I_9_ can be changed to the layer structured MA_3_Sb_2_I_9−*x*_Cl_*x*_ by HCl treatment.^23^ So far, the bandgaps of A_3_Sb_2_X_9_ perovskite materials have been narrowed by structure change from dimer to layer structure owing to the X site mixing of I and Cl. Here, we investigated if the bandgap of layer structured A_3_Sb_2_I_6_Cl_3_ mixed halide perovskite is controllable by A-site binary mixing of MA, FA, and Cs. Through the systematic studies on the effect of A-site binary mixing on its bandgap, we could find a layer structured FACs_2_Sb_2_I_6_Cl_3_ perovskite with narrower bandgap than the MA_3_Sb_2_I_6_Cl_3_ perovskite. In addition, we explained how the dual-site (A-site and X-site) mixed FACs_2_Sb_2_I_6_Cl_3_ perovskite has layer structure and relatively narrow bandgap by density functional theory (DFT) calculation.

To screen what binary combinations of A-site in A_3_Sb_2_I_9−*x*_Cl_*x*_ mixed halide perovskite can make narrower bandgap than the MA_3_Sb_2_I_6_Cl_3_ or Cs_3_Sb_2_I_6_Cl_3_ perovskite material with ∼2.4 eV of bandgap energy, we measured UV-visible absorption spectra and photographs of the A_3_Sb_2_I_9−*x*_Cl_*x*_ mixed halide perovskite films as shown in Fig. S1(a)–(d).† Apparently, the A_3_Sb_2_I_9−*x*_Cl_*x*_ mixed halide perovskite films had more red-shifted absorption spectra (Fig. S1(a)–(d)†) than the A_3_Sb_2_I_9-*x*_Br_*x*_ perovskites (Fig. S1(e) and (f)†). This implies that the A_3_Sb_2_I_9−*x*_Cl_*x*_ mixed halide perovskite films have 2D layer structures and the A_3_Sb_2_I_9-*x*_Br_*x*_ perovskites have 0D dimer structures. From the screening experiments, we chose the FACs_2_Sb_2_I_6_Cl_3_ perovskite material for further studies because it has the most red-shifted absorption spectrum as shown in Fig. S1(c).†

To compare optical properties of the previously reported dimer structured MA_3_Sb_2_I_9_ and layer structured MA_3_Sb_2_I_6_Cl_3_ and the A-sited mixed FACs_2_Sb_2_I_6_Cl_3_, we measured UV-visible absorption spectra in Fig. 1(a) and plotted corresponding Tauc plots in Fig. 1(b). The inset photographs in Fig. 1(a) indicate that the MA_3_Sb_2_I_9_, MA_3_Sb_2_I_6_Cl_3_, and FACs_2_Sb_2_I_6_Cl_3_ films have yellow, orange, and brown in color, respectively. The Tauc plots in Fig. 1(b) indicate that the bandgaps of MA_3_Sb_2_I_9_, MA_3_Sb_2_I_6_Cl_3_, and FACs_2_Sb_2_I_6_Cl_3_ films are 2.25 eV, 2.18 eV, and 2.0 eV, respectively. To check crystal structures of the MHP films, we measured X-ray diffraction (XRD) patterns of the MA_3_Sb_2_I_9_, MA_3_Sb_2_I_6_Cl_3_, and FACs_2_Sb_2_I_6_Cl_3_ and plotted the simulated XRD reference peaks of Cs_3_Sb_2_I_9_*P*6_3_/*mmc* 0D dimer structure, of which the Sb_2_I_9_^3−^ dimers share their triangular faces and form isolated structure,^17,24^ and Cs_3_Sb_2_I_9_*P*3̄*m*1 2D layer structure, of which the A-site cations act as spacers between the corner-sharing Sb_2_I_9_^3−^ octahedra,^17,24^ as shown in Fig. 1(c) and 2. Similarly to the UV-visible absorption spectra, the MA_3_Sb_2_I_6_Cl_3_ and FACs_2_Sb_2_I_6_Cl_3_ exhibited layer structures, whereas the MA_3_Sb_2_I_9_ had dimer structure. There were no additional peaks of impurities such as FACl, CsCl, and SbI_3_ in the XRD patterns. This indicates that the layer structured MHP films are formed by dual-site mixing.

**Fig. 1:**
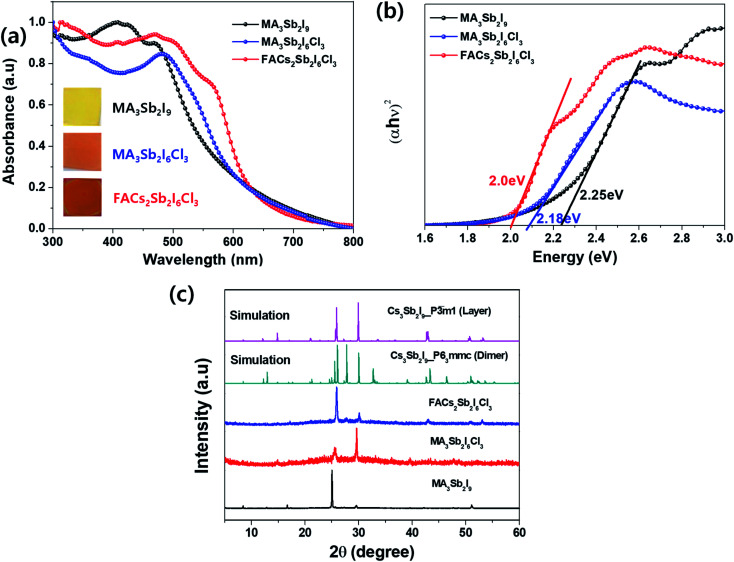
(a) UV-visible absorption spectra: inset = photographs of the MHP films, (b) Tauc plots, and (c) XRD patterns of MA_3_Sb_2_I_9_, MA_3_Sb_2_I_6_Cl_3_, and FACs_2_Sb_2_I_6_Cl_3_ MHP films.

**Fig. 2:**
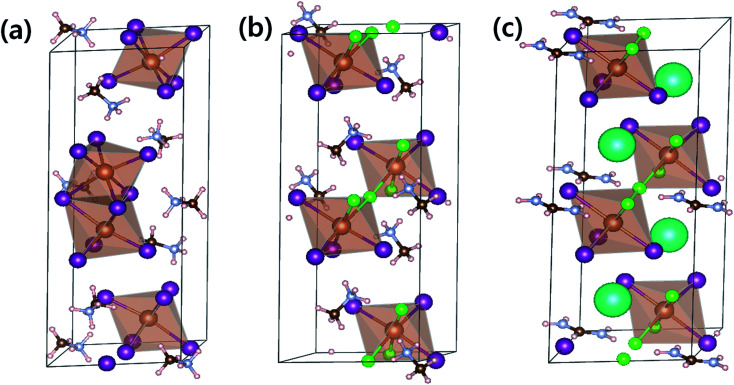
Lattice structures of (a) MA_3_Sb_2_I_9_ (*P*6_3_/*mmc*), (b) MA_3_Sb_2_I_6_Cl_3_ (*P*3̄*m*1), and (c) FACs_2_Sb_2_I_6_Cl_3_ (*P*3̄*m*1). Pink/brown/light gray/mint/light brown/green/purple spheres represent H/C/N/Cs/Sb/Cl/I respectively.

To understand the thermodynamic origins of dual-site mixing induced phase stabilization for the layered structures, we adopted density functional theory (DFT) using the Vienna *ab initio* simulation package (VASP).^25,26^ Details of DFT calculations are summarized in the ESI Section.† Optimized lattice structures for MA_3_Sb_2_I_9_ (*P*6_3_/*mmc*), MA_3_Sb_2_I_6_Cl_3_ (*P*3̄*m*1), and FACs_2_Sb_2_I_6_Cl_3_ (*P*3̄*m*1) are expressed in Fig. 2 and Table 1.

**Table 1:** Calculated lattice structures, volume per formula unit (FU), and calculated band gap with spin orbital coupling (SOC) and without SOC

	Calculated lattice structures	Calculated volume/FU Å^3^	Calculated band gap (eV)	
			PBEsol	PBEsol + SOC
MA_3_Sb_2_I_9_ (*P*6_3_/*mmc*)	*a* = 8.637 Å, *b* = 8.475 Å, *c* = 21.246 Å, *α* = 90.8°, *β* = 89.4° *γ* = 120.0°	673.4	1.82	1.69
MA_3_Sb_2_I_6_Cl_3_ (*P*3̄*m*1)	*a* = 8.077 Å, *b* = 8.362 Å, *c* = 10.233 Å, *α* = 89.9°, *β* = 90.8° *γ* = 119.05°	604.1	1.70	1.55
FACs_2_Sb_2_I_6_Cl_3_ (*P*3̄*m*1)	*a* = 8.145 Å, *b* = 8.403 Å, *c* = 9.888 Å, *α* = 91.1°, *β* = 91.1° *γ* = 118.7°	593.4	1.67	1.52

Band structures and density of states (DOS) are represented in Fig. 3(a–c). The 2D layer structured perovskites (*P*3̄*m*1) exhibit a more direct band gap like feature comparing with the 0D dimer structured perovskites (*P*6_3_/*mmc*). The calculated band gaps of the perovskites with and without spin orbital coupling (SOC) are summarized in Table 1. The FACs_2_Sb_2_I_6_Cl_3_ (*P*3̄*m*1) shows the smallest band gap regardless of SOC considering. The band gap narrowing by I–Cl mixed halide perovskites is attributed to the change of the lattice symmetry, *i.e.*, the phase change from *P*6_3_/*mmc* to *P*3̄*m*1.^17,22,27^ The reduced band gap for the FACs_2_Sb_2_I_6_Cl_3_ (*P*3̄*m*1) can be explained by its shorter bond length than the other perovskites. The volume per formula unit (FU) of FACs_2_Sb_2_I_6_Cl_3_ (*P*3̄*m*1) is the smallest even though their lateral dimension is slightly larger than MA_3_Sb_2_I_6_Cl_3_ (*P*3̄*m*1) as listed in Table 1. The ionic radius of Cs is the shortest among Cs, MA, and FA so that the volume of FACs_2_Sb_2_I_6_Cl_3_ can be the smallest. Accordingly, we can think that the FACs_2_Sb_2_I_6_Cl_3_ has the shortest bond length due to its smallest volume.

**Fig. 3:**
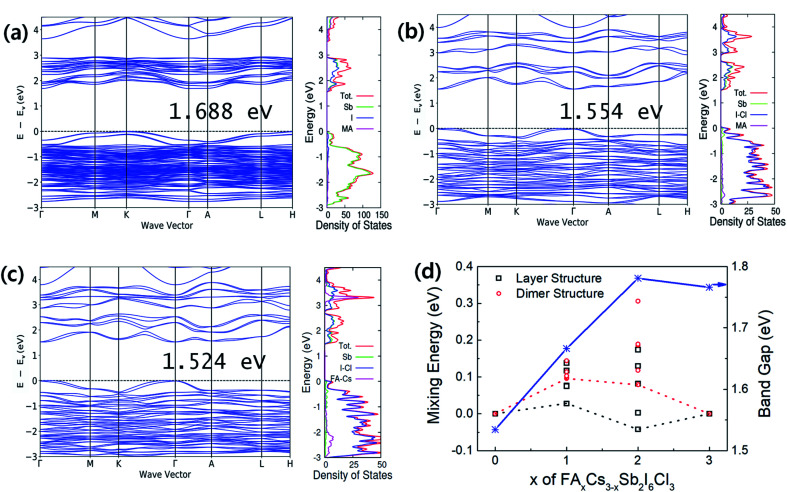
Band structures (left panel) and projected density of states (PDOS) of (a) MA_3_Sb_2_I_9_ (*P*6_3_/*mmc*), (b) MA_3_Sb_2_I_6_Cl_3_ (*P*3̄*m*1), and (c) FACs_2_Sb_2_I_6_Cl_3_ (*P*3̄*m*1). Valence band maximum energy is set to 0. Spin orbit coupling is included during the calculation. Calculated band gaps are underestimated than those of experimental values. (d) Solution energy changes by A-site mixing of FA_*x*_Cs_3−*x*_Sb_2_I_6_Cl_3_. The dotted lines are connecting the lowest energy states and dispersed symbols represent individual calculation result to obtain optimum lattice structures.

The role of A-site binary mixing on the stabilization of 2D layered structure is investigated by calculating solution energies as shown in Fig. 3(d). The solution energies of layered structures are smaller than those of dimer structures. The FACs_2_Sb_2_I_6_Cl_3_ (*P*3̄*m*1) has only 28 meV FU^−1^ of solution energy, which can be spontaneous reactions considering the contribution of configurational entropy.^28,29^ The DFT calculations clearly show that the dual-site mixing on A and X in A_3_Sb_2_X_9_ perovskite can be an efficient way to improve the stability of 2D layer structure and band gap modulation.

To check thermal properties of the dual-site mixed FACs_2_Sb_2_I_6_Cl_3_ MHP film, we checked thermal gravimetric analysis (TGA) thermogram and differential scanning calorimetry (DSC) as shown in Fig. 4(a) and (b). Here, we prepared the FACs_2_Sb_2_I_6_Cl_3_ MHP powder by mixing 1 : 2 : 2 molar ratio of FACl : CsCl : SbI_3_ instead of mixing 1 : 2 : 1 : 1 molar ratio of FAI : CsI : SbI_3_ : SbCl_3_ because the SbCl_3_ is more easily sublimed than the SbI_3_. The corresponding XRD patterns in Fig. S2† indicate that both samples have layered structures. The TGA and DTA (differential thermal analysis) spectra in Fig. 4(a) and S3† indicate that the FACl and SbI_3_ begins to be decomposed at ∼200 °C, whereas the CsCl starts to be decomposed at ∼700 °C. The prepared FACs_2_Sb_2_I_6_Cl_3_ MHP powder begins to be decomposed at ∼230 °C. This implies that the perovskite phase is formed by mixing of 1 : 2 : 2 molar ratio of FACl : CsCl : SbI_3_. The weight% of FACl : CsCl : SbI_3_ in FACs_2_Sb_2_I_6_Cl_3_ is 5.67 : 23.67 : 70.66 so the FACl and SbI_3_ are decomposed at 230–420 °C and the CsCl is decomposed over 420 °C. The higher decomposition temperature of prepared MHP powder at 150–300 °C than the pure FACl and the lower decomposition temperature of the powder at > 420 °C than the pure CsCl confirms that the new interaction bonding of FA–I and Cs–I is formed by formation of perovskite phase because their decomposition temperatures are FACl < FAI and CsI < CsCI in Fig. 4(a). Some I in the SbI_3_ might be replaced with Cl so its composition might be SbI_3−*x*_Cl_*x*_. Therefore, The FACs_2_Sb_2_I_6_Cl_3_ MHP powder might show an inflection point at ∼310 °C. The DSC spectrum in Fig. 4(b) shows that the synthesized FACs_2_Sb_2_I_6_Cl_3_ MHP powder begins to be decomposed at ∼230 °C, which is consistent with the TGA result.

**Fig. 4:**
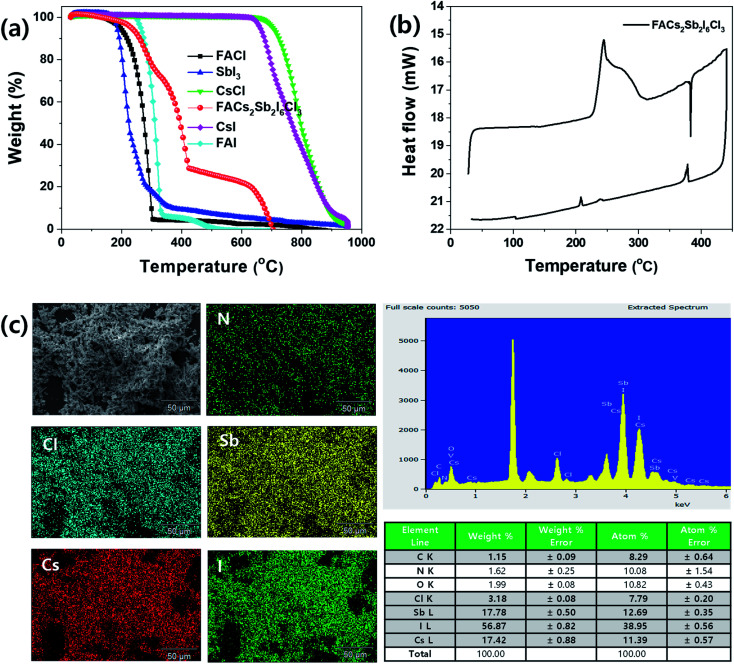
(a) Thermogravimetric analysis (TGA) spectra of FACl, SbI_3_, CsCl, CsI, FAI and FACs_2_Sb_2_I_6_Cl_3_ MHP powder, (b) differential scanning calorimeter (DSC) spectra of FACs_2_Sb_2_I_6_Cl_3_ MHP powder, and (c) energy disperse X-ray spectroscopy (EDS) elemental mapping and spectra of FACs_2_Sb_2_I_6_Cl_3_ MHP film.

Fig. 4(c) is energy dispersive X-ray spectroscopy (EDS) elemental mapping and spectrum of the synthesized FACs_2_Sb_2_I_6_Cl_3_ MHP powder. The Cl, Sb, Cs, and I elements were well dispersed in the EDS elemental mapping image so we can think that the perovskite phase is created. However, the EDS spectrum indicates that the formed FACs_2_Sb_2_I_6_Cl_3_ MHP powder is halide deficient because the molar ratio of Cs : Sb : I : Cl was 1.80 : 2.00 : 6.14 : 1.23. This might be attributed to the heat-treatment of the FACs_2_Sb_2_I_6_Cl_3_ MHP powder at 200 °C for 5 min under nitrogen atmosphere or the oxidation of SbI_3_. The UV-visible absorption spectra of the heat treated FACs_2_Sb_2_I_6_Cl_3_ MHP films at 110 °C, 150 °C, and 200 °C for 5 min under N_2_ atmosphere and photographs of corresponding films were shown in Fig. S4(a).† This clearly shows that the film is slightly darken by heat-treatment at 200 °C and its on-set absorption band edge is also slightly red-shifted. Their corresponding XRD patterns in Fig. S4(b)† indicate that their crystalline structures are maintained to 2D layer structure irrespective of heat-treatment temperature. The slightly narrowed XRD peaks by heat-treatment indicate that their crystallinities are slightly improved.

The electronic structure of synthesized FACs_2_Sb_2_I_6_Cl_3_ MHP film was analyzed by ultraviolet photoelectron spectroscopy (UPS) spectrum as shown in Fig. 5(a). The zoom-up UPS spectra confirm that the onset photoemission and valence band edge is 16.25 eV and 1.50 eV, respectively. Therefore, a calculated valence band maximum (VBM) energy (*E*_VBM_ = *hν* − *E*_cut-off_ + Δ*E*_VB_, where *hν* = 21.22 eV for He I, *E*_cut-off_ = 16.25 eV, and Δ*E*_VB_ = 1.50 eV) of the FACs_2_Sb_2_I_6_Cl_3_ MHP film is −6.47 eV. The calculated conduction band minimum (CBM) energy is −4.47 eV because its bandgap is 2.0 eV in Tauc plot. The band energy diagram of the TiO_2_ electrode based FACs_2_Sb_2_I_6_Cl_3_ MHP SC was shown in Fig. 5(b). The mesoscopic TiO_2_ based MHP SC was composed of FTO/bl-TiO_2_/*m*-TiO_2_/MHP/PTAA/Au. A representative SEM cross-sectional image of mesoscopic TiO_2_ based MHP SC was shown in Fig. 5(c). The SEM surface of MA_3_Sb_2_I_9_, MA_3_Sb_2_I_6_Cl_3_, and FACs_2_Sb_2_I_6_Cl_3_ film on *m*-TiO_2_/bl-TiO_2_/FTO substrate were shown in Fig. 5(d–f), respectively. The current density–voltage (*J*–*V*) curves of mesoscopic MA_3_Sb_2_I_9_, MA_3_Sb_2_I_6_Cl_3_, and FACs_2_Sb_2_I_6_Cl_3_ MHP SCs in Fig. 5(g) indicate that the dual-site mixed FACs_2_Sb_2_I_6_Cl_3_ MHP SC have the highest PCE than the other MHP SCs. A short-circuit current density (*J*_sc_), open-circuit voltage (*V*_oc_), fill factor (FF), and PCE of FACs_2_Sb_2_I_6_Cl_3_ MHP SC was 2.88 mA cm^−2^, 0.59 V, 61.6%, and 1.05% at 1 sun condition. Their photovoltaic parameters were summarized in Table 2. Average photovoltaic properties of 16 samples for each device were shown in Fig. S5.† The FACs_2_Sb_2_I_6_Cl_3_ MHP SC did not show significant J-V hysteresis with respect to the scan direction as shown in Fig. S6.† Their external quantum efficiency (EQE) spectra were shown in Fig. 5(h). Although their PCEs are still very poor, it is clear that the dual site-mixed A_3_Sb_2_X_9_ MHP SCs have 2D layer structure and narrower bandgap. To check the stabilities of MA_3_Sb_2_I_9_, MA_3_Sb_2_I_6_Cl_3_, and FACs_2_Sb_2_I_6_Cl_3_ MHP SCs, we tracked the maximum power points (MPPs) of un-encapsulated devices for 60 min under continuous light soaking at 1 sun as shown in Fig. 5(i). This clearly shows that the FACs_2_Sb_2_I_6_Cl_3_ MHP SC has good stability.

**Fig. 5:**
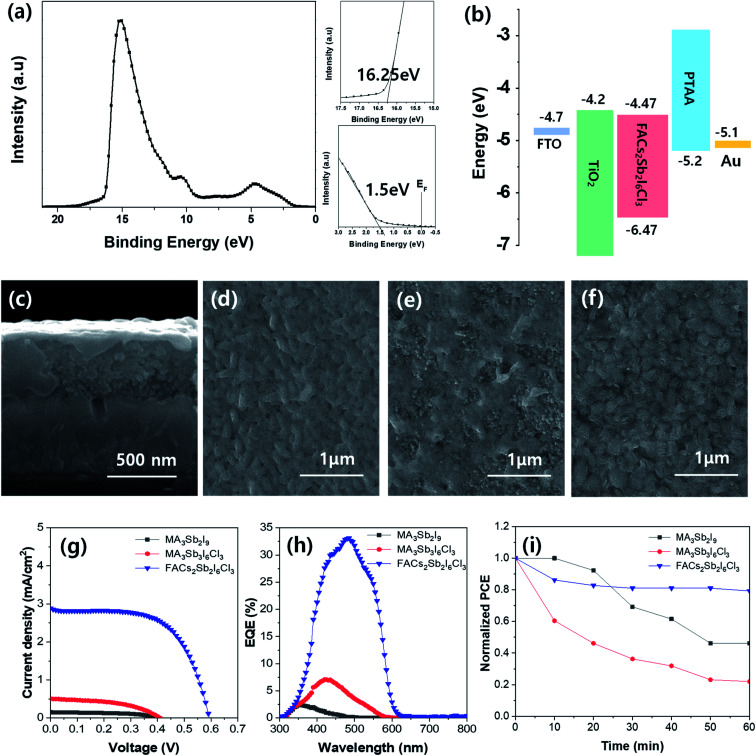
(a) Ultraviolet photoelectron spectroscopy (UPS) spectrum of FACs_2_Sb_2_I_6_Cl_3_ MHP film, (b) energy band diagram of MHP SC, (c–f) representative SEM cross-sectional image of MHP SC (c) and SEM surface images (d–f) of FTO/bl-TiO_2_/*m*-TiO_2_/MHP ((d) MA_3_Sb_2_I_9_, (e) MA_3_Sb_2_I_6_Cl_3_, and (f) FACs_2_Sb_2_I_6_Cl_3_), and (g–i) photovoltaic properties of MHP SCs: (g) current density–voltage curves, (h) EQE spectra, and (i) stabilities of mesoscopic MA_3_Sb_2_I_9_, MA_3_Sb_2_I_6_Cl_3_, and FACs_2_Sb_2_I_6_Cl_3_ MHP SCs and planar FACs_2_Sb_2_I_6_Cl_3_ MHP SC.

**Table 2:** Summary of photovoltaic parameters of mesoscopic MA_3_Sb_2_I_9_, MA_3_Sb_2_I_6_Cl_3_, and FACs_2_Sb_2_I_6_Cl_3_ MHP SCs

Device	*J*_sc_ (mA cm^−2^)	*V*_oc_ (V)	FF (%)	PCE (%)
MA_3_Sb_2_I_9_	0.162	0.40	55.0	0.03
MA_3_Sb_2_I_6_Cl_3_	0.51	0.42	47.9	0.10
FACs_2_Sb_2_I_6_Cl_3_	2.88	0.59	61.6	1.05

## Conclusions

In summary, we could synthesize 2D layer structured FACs_2_Sb_2_I_6_Cl_3_ perovskite with narrower energy bandgap by dual-site mixing. The narrower bandgap of FACs_2_Sb_2_I_6_Cl_3_ perovskite than the 0D dimer structured MA_3_Sb_2_I_9_ and the 2D layer structured MA_3_Sb_2_I_6_Cl_3_ is attributed to the phase change from *P*6_3_/*mmc* dimer structure to *P*3̄*m*1 layer structure due to the change of lattice symmetry by I–Cl mixed halide and the shorter bond length than the others due to the shortest ionic radius of Cs among Cs, MA, and FA. Consequently, the dual-site mixed FACs_2_Sb_2_I_6_Cl_3_ MHP SCs exhibited higher PCE of 1.05% at 1 sun conditions than the dimer structured MA_3_Sb_2_I_9_ MHP SCs and the layer structured MA_3_Sb_2_I_6_Cl_3_ MHP SCs.

## Conflicts of interest

There are no conflicts to declare.

## Supplementary Material

RA-010-D0RA00787K-s001
