# Supplementary material for: Dual-site mixed layer-structured FAxCs3−xSb2I6Cl3 Pb-free metal halide perovskite solar cells
Source: RSC Adv. 2020 May 6;10(30):17724–30. doi: 10.1039/d0ra00787k (PMC9053595; doi:10.1039/d0ra00787k)
Supplement: RA-010-D0RA00787K-s001 [file RA-010-D0RA00787K-s001.pdf]

Electronic Supplementary Material (ESI) for RSC Advances.
This journal is © The Royal Society of Chemistry 2020

Electronic Supplementary Information (ESI)

Dual-site mixed layer-structured $FA_xCs_{3-x}Sb_2I_6Cl_3$ Pb-free metal halide perovskite solar cells

Yong Kyu Choi,[a] Jin Hyuck Heo,[a] Ki-Ha Hong,*[,b] Sang Hyuk Im,*[,a]

[a.] *Department of Chemical and Biological Engineering, Korea University, 145 Anam-ro, Seongbuk-gu, Seoul 02841, Korea. E-mail: imromy@korea.ac.kr*

[b.] *Department of Materials Science and Engineering, Hanbat National University, 125 Dongseo-daero, Yuseong-Gu, Daejeon, 34158, Korea. E-mail: kiha.hong@hanbat.ac.kr*

Experimental Section

Materials

All reagents were purchased from the following companies and used without further purifications. CsI (Cesium iodide, 99.9% Sigma-Aldrich), SbI_3 (antimony(lll) iodide, 99.8% Sigma-Aldritch), $SbCl_3$ (Antimony(III) chloride, 99% Sigma-Aldrich), CsCl (Cesium Chloride, 99.9%, Sigma-Aldrich), HI solution (hydroiodic acid, 57wt% in H_2O, Sigma-Aldrich), FACl (Formamidinium Chloride, >97%, TCI), formamidinium acetate salt (99%, Sigma-Aldrich), methylamine solution (40 wt.% in H_2O, Sigma-Aldrich), anhydrous ethanol (99.9%, Samchun), diethyl ether (99.0%, Samchun), N,N dimethylformamide DMF, Sigma-Aldrich), titanium diisopropoxide bis(acetylacetate) (TAA, Sigma-Aldrich), Li-bis(trifluoromethanesulfonyl) imide (Li-TFSI, Sigma-Aldrich), tert-butylpyridin (t-BP, Sigma-Aldrich), acetonitrile (Sigma-Aldrich), and poly-triarylamine (PTAA, EM-index) were purchased.

Synthesis of formamidinium iodide ($HC(NH_2)_2I$, FAI)

FAI was synthesized by dissolving 17.9g of formamidinium acetate salt in 10 ml of anhydrous ethanol charged in round bottomed flask (RBF) and the solution was cooled to below 0 ^oC in an ice bath. Then, 25 ml of HI solution was slowly added by dropping funnel into the solution for 2 h. The solution was dried by a rotary evaporator. The powder was purified by recrystallization in diethyl ether. The recrystallized powder was then dried overnight in a vacuum oven.

Synthesis of methylammonium iodide ($(CH_3NH_3)I$, MAI)

MAI was synthesized by dissolving 14.8 ml of methylamine solution in 10 ml of anhydrous ethanol charged in round bottomed flask (RBF) and the solution was cooled to below 0 ^oC in an ice bath. Then, 25 ml of HI solution was slowly added by dropping funnel into the solution for 2 h. The solution was dried by a rotary evaporator. The powder was purified by recrystallization in diethyl ether. The recrystallized powder was then dried overnight in a vacuum oven.

Synthesis of methylammonium Chloride ((CH$_3$NH$_3$)Cl, MACl)

MACl was synthesized by dissolving 23.8 ml of methylamine solution in 10 ml of anhydrous ethanol charged in round bottomed flask (RBF) and the solution was cooled to below 0 ^{o}C in an ice bath. Then, 25 ml of HCl solution was slowly added by dropping funnel into the solution for 2 h. The solution was dried by a rotary evaporator. The powder was purified by recrystallization in diethyl ether. The recrystallized powder was then dried overnight in a vacuum oven.

Preparation of $MA_3Sb_2I_9$, $MA_3Sb_2I_6Cl_3$, and $FACs_2Sb_2I_6Cl_3$ MHP solutions

For the preparation of $MA_3Sb_2I_9$, $MA_3Sb_2I_6Cl_3$, and $FACs_2Sb_2I_6Cl_3$ solution, we prepared 0.3M MAI, 0.3M MACl, 0.3M FACl, 0.3M CsCl, and 0.3M SbI_3 in DMF. We mixed 3:2 (vol.:vol.) of MAI:SbI_3 solution for $MA_3Sb_2I_9$, 3:1 (vol.:vol.) of MACl:SbI_3 solution for $MA_3Sb_2I_6Cl_3$, and 1:2:2 (vol.:vol.:vol.) of FACl:CsCl:SbI_3 solution were for $FACs_2Sb_2I_6Cl_3$. To make a clear solution by enhancing solubility, we added HI solution in the MHP solution (HI:MHP solution = 1:100 vol.:vol.).

Fabrication of MHP SCs

For MHP SCs, ~50 nm-thick blocking TiO_2 (bl-TiO_2) was deposited on a patterned FTO substrate (Pilkington, TEC-8) by a spray pyrolysis deposition using 20 mM of TAA solution at 500 ^{o}C. A mesoporous TiO_2 (m-TiO_2, Share-Chem) with ~400 nm-thickness was deposited by spin-coating on the bl-TiO_2/FTO substrate at 5000 rpm for 30 s and calcined at 500 °C for 1 h in air to remove organic components. For the fabrication of MHP films, the m-TiO_2/bl-TiO_2/FTO substrates were treated by argon atmospheric pressure plasma (FemtoScience). Then the MHP solutions filtered by 0.45 μm polytetrafluoroethylene (PTFE) were spin-coated on the mp-TiO_2/bl-TiO_2/FTO substrates at 2000 rpm for 30 s. During the spin-coating process, cold diethyl ether wad dropped on the spinning substrates in ~10 s after beginning spin-coating. The MHP/ mp-TiO_2/bl-TiO_2/FTO substrates were then heat-treated on a hotplate at 200 °C for 5 min at N_2 atmosphere. PTAA hole transporting layer was formed by spin-coating of PTAA/toluene (15 mg/1 ml) with 7.5 μL of Li-TFSI/acetonitrile (170 mg/1 mL) and 7.5 μl of t-BP/acetonitrile (1 mL/1 mL) additives on the MHP/ mp-TiO_2/bl-TiO_2/FTO substrates at 5000 rpm for 30 s. Finally, an Au counter electrode was deposited by thermal evaporation. The active areas of all devices were fixed to 16 mm^2.

Characterizations

The morphologies of the prepared samples were characterized using a field-emission scanning electron microscope (FE-SEM, FEI, Quanta 250 FEG). The crystal structure of the obtained product was examined by an X-ray diffractometer (XRD, Smartlab, Rigaku). The changes in the work-function and highest occupied molecular orbital (HOMO) states were measured by an ultra violet photoelectron spectroscopy (UPS, Thetaprobe (Thermo)). The external quantum efficiency (EQE) was measured by a power source (ABET, 150 W xenon lamp, 13014) with a monochromator (Dongwoo Optron Co., Ltd., MonoRa500i) and a potentiostat (Ivium, IviumStat). The current density-voltage (J-V) curves were measured by a solar simulator (Peccell, PEC-L01) with a potentiostat (IVIUM, IviumStat) under an illumination of 1 sun (100 mW/cm^2 AM 1.5G) and a certified Si-reference

cell from JIS (Japanese Industrial Standards). The J-V curves of all devices were measured by masking the active area with a metal mask (aperture = 9.6 mm^2).

DFT calculations

Density function theory (DFT) calculations were conducted with the Vienna ab initio simulation package (VASP).[S1,S2] We adopted the modified the modified Perdew–Burke–Ernzerhof generalized gradient approximation (PBEsol-GGA).[S3] which has been successfully applied to reproduce the experimental lattice constants of halide perovskites.[S4,S5] Valence electronic wave functions are expanded in a planewave basis set with a cutoff energy of 500 eV. The optimized lattice structures are obtained by fully relaxing atomic movement and lattice vectors using Γ-centered k-point grid by $4 \times 4 \times 4$ and $4 \times 4 \times 2$ for layered and dimer structures, respectively. We calculated minimally five atomic configurations for mixed perovskites and then select the minimum energy configuration.

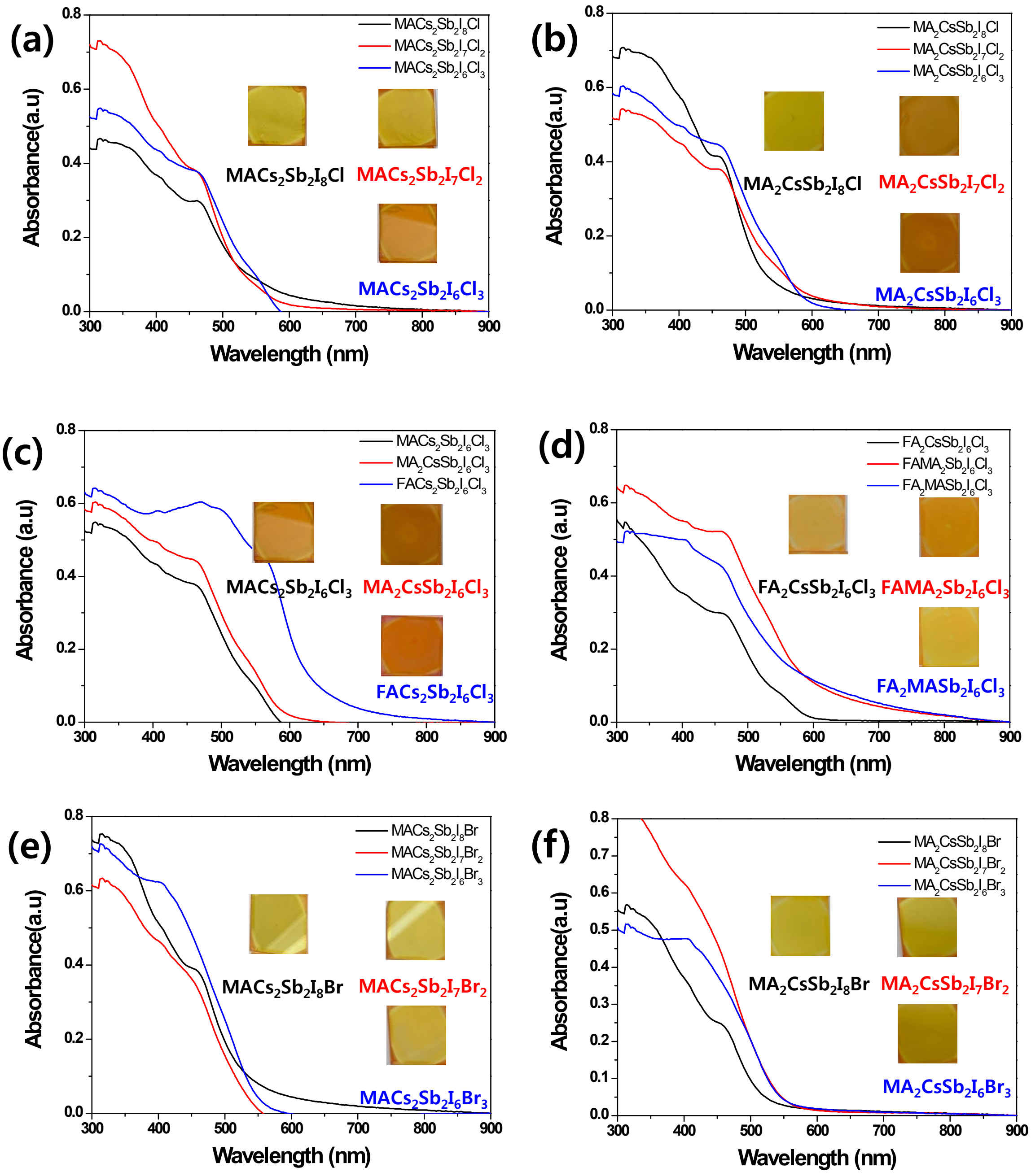

Figure S1. Screening experiments to find narrow bandgap of dual-sited mixed MHP films. (a) $MACS_2Sb_2I_6Cl_3$, $MACs_2Sb_2I_7Cl_2$, and $MACs_2Sb_2I_8Cl$ film, (b) $MA_2CsSb_2I_6Cl_3$, $MA_2CsSb_2I_7Cl_2$, and $MA_2CsSb_2I_8Cl$ film, (c) $MACs_2Sb_2I_6Cl_3$, $MA_2CsSb_2I_6Cl_3$, and $FACs_2Sb_2I_6Cl_3$ film, (d) $FA_2CsSb_2I_6Cl_3$, $FAMA_2Sb_2I_6Cl_3$, and $FA_2MASb_2I_6Cl_3$ film, (d) $MACs_2Sb_2I_8Br$, $MACs_2Sb_2I_7Br_2$, and $MACs_2Sb_2I_6Br_3$ film, and (f) $MA_2CsSb_2I_8Br$, $MA_2CsSb_2I_7Br_2$, and $MA_2CsSb_2I_6Br_3$ film.

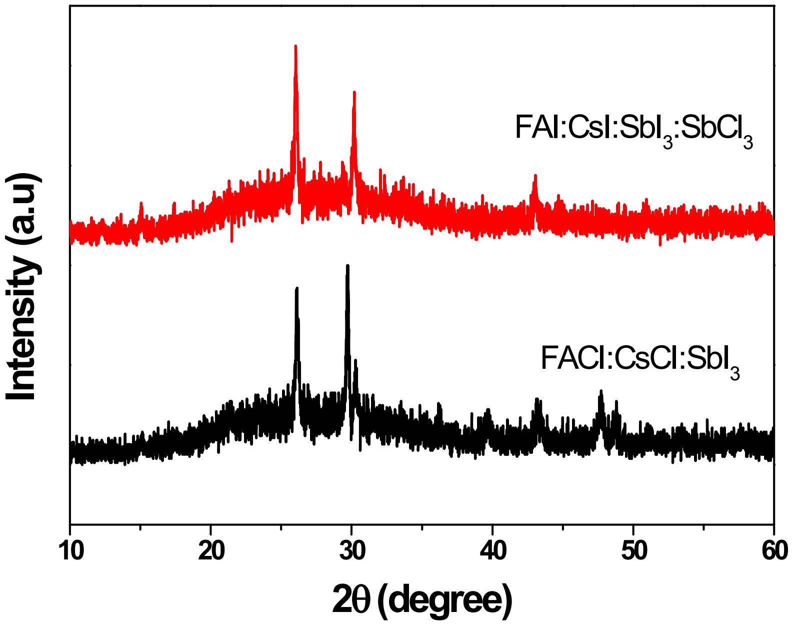

Figure S2. XRD patterns of $FACs_2Sb_2I_6Cl_3$ MHP powder prepared by mixing 1:2:2 molar ratio of FACl:CsCl:SbI_3 (black line) and 1:2:1:1 molar ratio of FAI:CsI:SbI_3:$SbCl_3$ (red line).

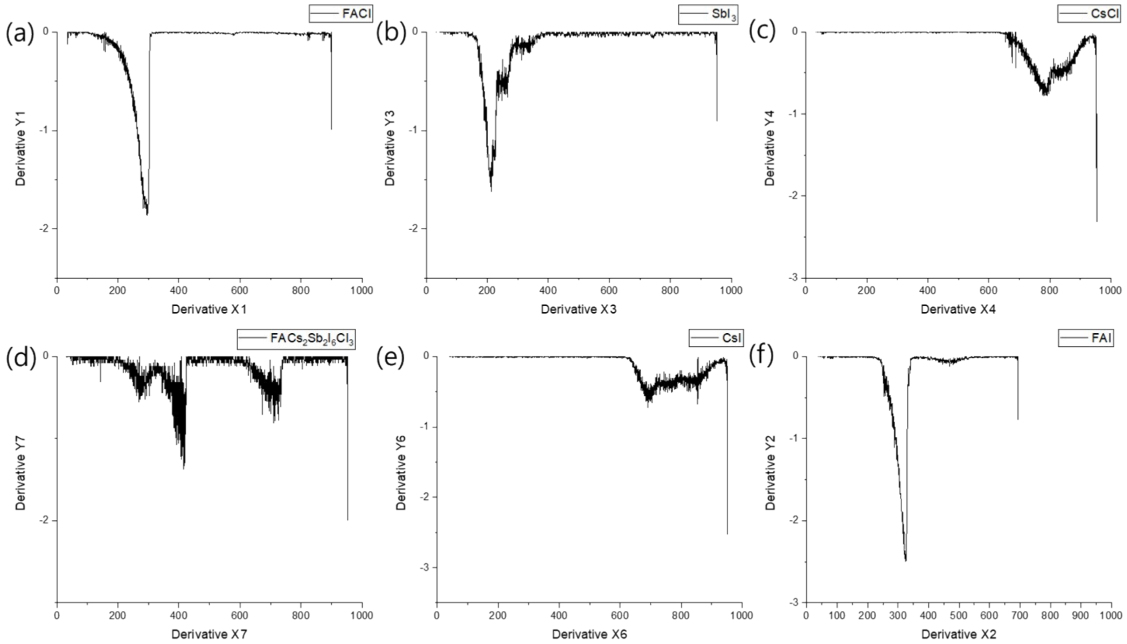

Figure S3. DTA (differential thermal analysis) spectra of each component of (a) FACl, (b) SbI_3, (c) CsCl, (d) $FACS_2Sb_2I_6Cl_3$, (e) CsI, and (f) FAI.

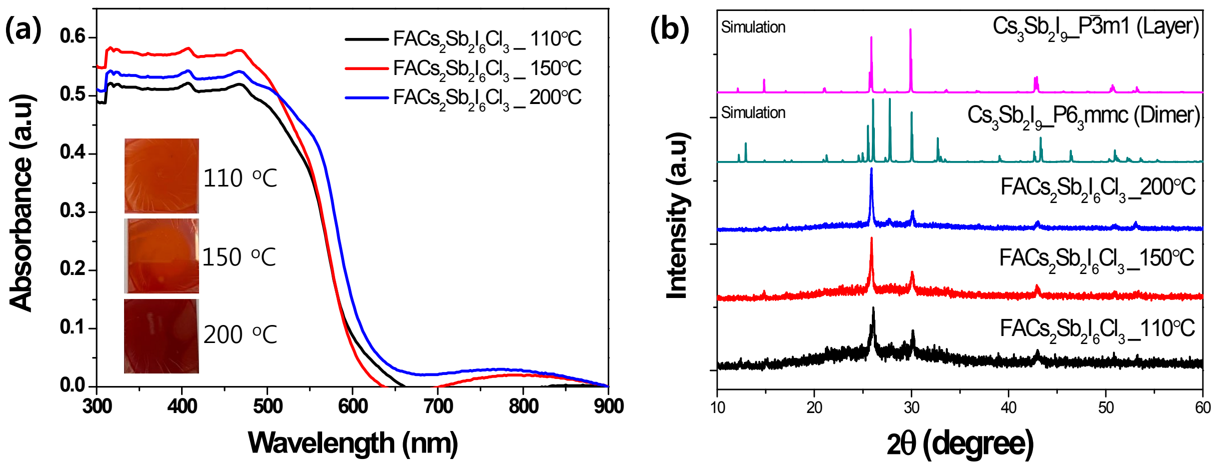

Figure S4. (a) UV-visible absorption spectra (inset = photographs of the heat-treated MHP films) and (b) XRD patterns of $FACs_2Sb_2I_6Cl_3$ MHP films heat-treated at different temperatures.

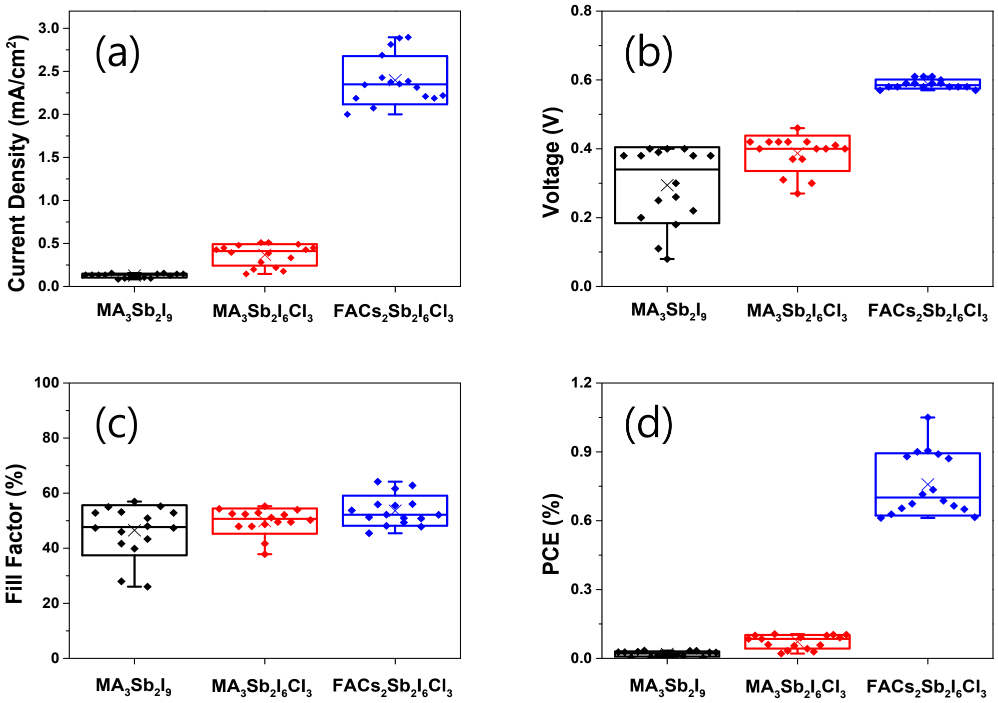

(e)

Device	J_{sc} (mA/cm^2)	V_{oc} (V)	FF (%)	PCE (%)
$MA_3Sb_2I_9$	0.12±0.14	0.29±0.01	46.53±4.70	0.02±0.01
$MA_3Sb_2I_6Cl_3$	0.36±0.06	0.38±0.06	49.86±2.36	0.07±0.01
$FACs_2Sb_2I_6Cl_3$	2.40±0.14	0.58±0.01	53.61±2.82	0.76±0.07

Figure S5. Average photovoltaic properties of 16 samples for each device: (a-d) box plots of current density (a), voltage (b), fill factor (c), and PCE (d) and their summaries (e).

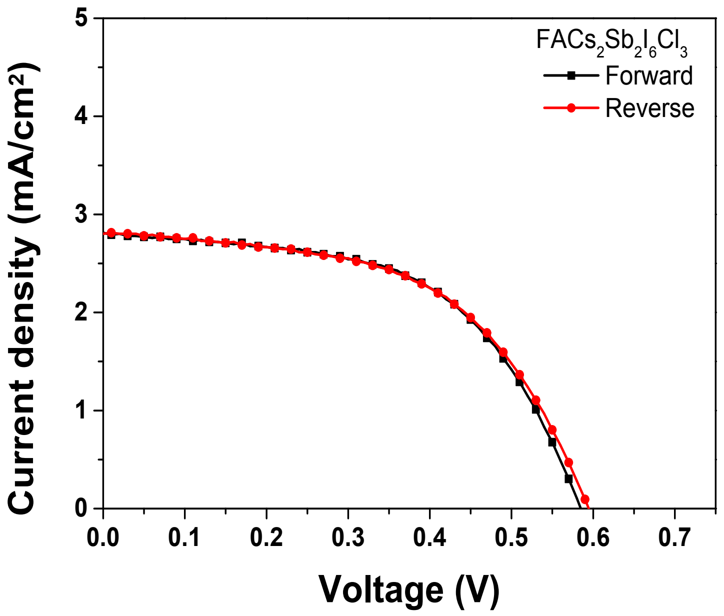

Figure S6. J-V curves of $FACs_2Sb_2I_6Cl_3$ MHP SC measured by forward and reverse scan condition.

References

(S1) Kresse, G.; Hafner, J. Ab Initio Molecular Dynamics for Liquid Metals. *Phys. Rev. B.* **1993.** *47*, 558–561

(S2) Kresse, G.; Furthmüller, J. Efficiency of Ab-Initio Total Energy Calculations for Metals and Semiconductors Using a Plane-Wave Basis Set. *Comput. Mater. Sci.* **1996**. *6,* 15–50

(S3) Perdew, J. P.; Ruzsinszky, A.; Csonka, G. I.; Vydrov, O. A.; Scuseria, G. E.; Constantin, L. A.; Zhou, X.; Burke, K.; Restoring the Density-Gradient Expansion for Exchange in Solids and Surfaces. *Phys. Rev. Lett.* ***2008,*** *100*, 136406.

(S4) Kim, J., Lee, S.-H., Chung, C.-H.; Hong, K.-H. Systematic Analysis of the Unique Band Gap Modulation of Mixed Halide Perovskites. *Phys. Chem. Chem. Phys.* **2016,** *18*, 4423–4428

(S5) Leguy, A. M. A.; Frost, J. M.; Mcmahon, A. P.; Sakai, V. G.; Kockelmann, W.; Law, C.; Li, X.; Foglia, F.; Walsh, A.; O'Regam, B. C.; Nelson, J.; Cabral, J. T.; Barnes, P. R. F.; The Dynamics of Methylammonium Ions in Hybrid Organic-Inorganic Perovskite Solar Cells. *Nat. Com.* **2015,** *6,* 7124
